# Molecular mechanisms and translational potential of plant-derived nanovesicles in skin care and regeneration

**DOI:** 10.3389/fcell.2026.1877038

**Published:** 2026-08-28

**Authors:** Lele Wang, Xiang Li, Mengke Sheng, Yue Li, Shaobo Liu, JiaYing Wu, Hu Huang, Hang Fan

**Affiliations:** 1 Henan Academy of Sciences, Zhenzhou, China; 2 Henan High-tech Industrial Co., Ltd., Zhenzhou, China; 3 Proya Cosmetics Co., Ltd, Hangzhou, China; 4 Zhongyuan Meigu Technology Industry Co., Ltd., Luoyang, China

**Keywords:** antioxidant, drug delivery, nanocarrier, plant-derived nanovesicles (PDNVs), skin care

## Abstract

Plant-derived nanovesicles (PDNVs) are emerging as a novel class of natural biomaterials with significant potential in dermatological applications, owing to their unique lipid bilayer structure, broad source availability, and low immunogenicity. This review systematically delineates the biogenesis pathways of PDNVs—including the multivesicular body (MVB) and exocyst-positive organelle (EXPO) routes—and characterizes their complex cargo, which encompasses functional proteins, regulatory microRNAs, and bioactive secondary metabolites. We critically discuss the molecular mechanisms underlying the skincare efficacy of PDNVs, emphasizing their roles in antioxidant defense via Nrf2 activation, anti-aging effects through the modulation of MMP expression and collagen synthesis, melanogenesis inhibition, and the promotion of wound healing by regulating inflammatory cytokines and the TGF-β pathway. Furthermore, the article compares PDNVs with synthetic nanocarriers and other extracellular vesicles, highlighting their superior biocompatibility and cellular uptake efficiency as natural delivery systems. Finally, we address current industrial challenges related to standardized isolation, quality control, and storage stability, and propose future perspectives on bioengineering strategies and clinical translation. This work provides a comprehensive theoretical foundation for the application of PDNVs in precision dermatology and cosmetic biotechnology.

## 1 Introduction

As the largest organ of the human body, the skin serves as the primary physical barrier against external environmental insults and a crucial indicator of overall health status. With the improvement of living standards and the heightened pursuit of aesthetic appeal, skin care has progressively evolved into a critical component of health management. Recent advancements in nanobiotechnology have revealed the considerable potential of plant-derived nanovesicles (PDNVs), a novel class of nanoscale biomaterials, in the field of skin care.

Extracellular vesicles (EVs) originate from both eukaryotic and prokaryotic cells. They are small cell-derived vesicles. In addition to proteins and lipids, EVs contain nucleic acids, namely, mRNA and miRNA. These components mediate intercellular communication in various biological processes (Urbanelli et al., 2013; Brown et al., 2015; Xie et al., 2022). The International Society for Extracellular Vesicles (ISEV) updated the definition of EVs in 2024. According to this definition, EVs are particles released from cells. They are separated by a lipid bilayer. Additionally, they are incapable of self-replication. Specifically, they do not contain a functional nucleus. (Hao et al., 2022). Animal extracellular vesicles (EVs) have sparked a surge of research due to their pivotal roles in intercellular communication and information transfer, disease diagnosis and monitoring, disease treatment, and immune regulation. Mammalian EVs can be classified based on their cellular origins, such as cell culture media, serum, blood, urine, and other bodily fluids. In contrast, plant EVs cannot be further subclassified according to subcellular origins. In response to this, the International Society for Extracellular Vesicles (ISEV) proposes that all vesicular components derived from plant tissues be termed “plant-derived nanovesicles” (PDNVs) (Pinedo et al., 2021a), encompassing those released from cells as well as those obtained through destructive processes where natural release into the extracellular space cannot be confirmed.

Research on plant-derived vesicles has evolved significantly. As early as 1967, before the discovery of animal extracellular vesicles, researchers had already observed through electron microscopy that plant cells, specifically *Daucus carota* cells, could secrete vesicles (Halperin and Jensen, 1967). Over the past 15 years, studies have revealed the crucial roles of PDNVs in plant material transport, signal transduction, and immune defense. In MLA12-barley (*Hordeum vulgare*) infected with *Blumeria graminis f. Sp. Hordei*, multivesicular bodies and vacuolar invaginations were found to proliferate and accumulate in both infected epidermal cells and uninfected cells. All plasmodesmata were contracted or blocked by cell wall appendages, thereby preventing fungal penetration and reducing tissue damage (An et al., 2006). Research has revealed that *Arabidopsis thaliana* cells secrete nanovesicles capable of transporting small RNAs (sRNAs) into fungal cells, where they silence critical pathogenicity genes to exert a defensive mechanism (Cai et al., 2018). Further, Zhao (Zhao et al., 2018) employed real-time fluorescent quantitative PCR to detect microRNAs in nanovesicles isolated from both mature and immature coconut milk, confirming that these vesicles facilitate, the growth and maturation process of c*ocos nuciferas.* Over the past 15 years, research on PDNVs has been significantly refined. This advancement is driven by findings that suggest their potential significance in multiple areas. These areas include plant material transport, signal transduction, immune defense, and interactions with other organisms. Consequently, PDNVs present a vast array of application prospects.

Compared to animal-derived exosomes, PDNVs offer distinct advantages, including broad source availability, cost-effectiveness, scalability for mass production, low immunogenicity, and excellent biocompatibility, making them a research focus in skin care. PDNVs not only encapsulate diverse plant-derived bioactive compounds, such as polyphenols, terpenoids, and polysaccharides, but also possess unique nanostructures and biological functions that enable efficient penetration of the skin barrier, thereby exerting multiple therapeutic effects, including antioxidant, anti-inflammatory, anti-aging, and wound healing activities. Beyond the vesicles themselves, the intrinsic phytochemical profiles of the source plants are fundamental to their bioactivity. For instance, essential oils derived from aromatic plants such as *Rosmarinus officinalis* (rosemary) are rich in polyphenolic diterpenes (e.g., carnosic acid and rosmarinic acid), which contribute significantly to antioxidant and anti-aging effects in dermocosmetic formulations (Diass et al., 2023; Achagar et al., 2024).

This review systematically elucidates the research progress in PDNVs for skin care over the past decade, focusing on both fundamental research and translational applications (Cheng et al., 2025; Gu et al., 2025; Ramírez et al., 2025; Sun et al., 2025b; Sun et al., 2025 M.; Wang J. et al., 2025; Wei et al., 2025; Zheng et al., 2025). Furthermore, we present a comparative analysis of the advantages and limitations of PDNVs relative to synthetic nanocarriers and animal-derived exosomes, aiming to provide a theoretical foundation for future research and application development in skin care.

## 2 Characteristics, biogenesis and compositions of PDNVs

### 2.1 Characteristics of PDNVs

PDNVs are nanoscale membranous vesicles actively secreted by plant cells. They are characterized by a typical lipid bilayer structure. Additionally, they often exhibit a cup-shaped or hemispherical morphology. Compared to their animal-derived counterparts, PDNVs exhibit a broader size distribution, typically ranging from 50 to 300 nm, with an average diameter of approximately 150–200 nm. Key physicochemical parameters for characterizing PDNVs include particle concentration, zeta potential (which indicates surface charge and colloidal stability), and morphological integrity (Table 1). These characteristics are significantly influenced by the plant source, extraction methods, and growth conditions, leading to substantial variability among PDNVs from different species (Teng et al., 2025).

**TABLE 1:** Physicochemical characterization of representative PDNVs.

Source	Diameter/nm	Zeta potential/mV	Appearance	Ref
*Citrus paradisi*	210.8	−49.2 to −1.52	Double-layer membrane structure with smooth surface	(Wang et al., 2014)
*Triticum aestivum*	75	−	Spherical particles, double membrane structure	(Beit-Yannai et al., 2019)
Shiitake Mushroom	126.7 ± 11.3	−	Sphere-shaped morphology	(Liu et al., 2020)
*Dendropanax morbifera*	90	−	Nearly spherical, typical bilayer structure	(Lee et al., 2020a)
*Zea mays*	80	−17	Cavity structure with double membrane appearance	(Sasaki et al., 2021)
*Zingiber officinale*	156 ± 36	−26.6 ± 5	Bilamellar appearance, exhibiting a cupular structure	(Yin et al., 2022)
Mullberry Bark	151.3 ± 45.4	−	Typical bilayer membrane structure	(Sriwastva et al., 2022)
*Catharanthus roseus*	75.51 ± 10.19	−21.8	Round nanoparticles	(Ou et al., 2023)
*Camellia sinensis*	166.9	−28.8	Spherical pellet	(Zhao et al., 2023)
*Panax notoginseng*	151.3	8	spherical, and exhibit a cup-shaped structure	(Li et al., 2023)
*Houttuynia cordata*	169.5	−23.04 ± 1.22	Double-layer membrane structure with uniform size	(Zhu et al., 2023)
Spinach	135.6	−5–6	Spherical nanoparticles, bilayer structure	(Lee et al., 2024)

### 2.2 Biogenesis of PDNVs

The biogenesis of PDNVs involves multiple pathways within plant cells, contributing to the diversity of vesicle populations observed in plant extracts. The primary pathways include the vacuolar pathway, where vesicles bud from the tonoplast; the multivesicular body (MVB) pathway, where intraluminal vesicles are formed within MVBs and released upon fusion with the plasma membrane; and the exocyst-positive organelle (EXPO) pathway, which mediates unconventional secretion. The relative contribution of each pathway varies depending on the plant species, cell type, and physiological or stress conditions (Mu et al., 2023).

The MVB pathway is considered the primary mechanism for PDNV generation. It has been well characterized in plant cells. The pathway centers on multivesicular bodies (MVBs). These MVBs originate from endocytosis. This endocytosis is initiated by plasma membrane invagination (Alenquer and Amorim, 2015). Following invagination, early endosomes are formed. These early endosomes subsequently mature into late endosomes through interactions with the trans-Golgi network (TGN). MVBs are defined by the presence of intraluminal vesicles (ILVs), which are 30–100 nm in diameter. The ILVs selectively package diverse cytoplasmic components, including lipids, proteins, nucleic acids, and small molecules (Li et al., 2018). The biogenesis of ILVs is mediated by the Endosomal Sorting Complex Required for Transport (ESCRT) machinery, which facilitates membrane curvature and scission. Finally, the fusion of MVBs with the plasma membrane occurs. This fusion results in the release of their ILVs as extracellular vesicles (EVs) in mammalian systems. In the context of plant biology, however, these entities are referred to as plant-derived nanovesicles (PDNVs) to avoid assumptions regarding conserved biogenesis pathways. These PDNVs are functional, uniformly-sized vesicles characterized by their endosomal origin. They can subsequently enter recipient cells via receptor-mediated endocytosis or direct membrane fusion to deliver their bioactive cargo.

In addition to the canonical MVB pathway, plant-specific pathways also contribute to PDNV secretion. One such mechanism is the EXPO pathway, which represents a plant-specific secretory mechanism (Wang et al., 2010). EXPOs are double-membraned, spherical organelles originally identified in *Arabidopsis thaliana* and structurally resemble autophagosomes. Despite their structural similarity to autophagosomes, EXPOs are functionally distinct. They fuse with the plasma membrane to release single-membrane vesicles-now widely designated as plant-derived nanovesicles (PDNVs) or exosome-like nanoparticles (PELNs)-into the apoplast or cell wall space. The EXPO secretory pathway comprises three key steps: (1) the intracellular biogenesis of EXPOs, (2) their targeted fusion with the plasma membrane, and (3) the subsequent release of PDNVs (Cui et al., 2019). Key marker proteins, including Exo70H1 and Exo70B2, play critical roles in EXPO-mediated trafficking. These proteins are not only associated with plant innate immunity but also directly induce pathogen-triggered immunity (PTI), thereby enhancing Arabidopsisresistance to pathogens. Consequently, vesicles derived from the EXPO pathway serve as important mediators of intercellular communication and are instrumental in transducing plant defense signals.

**FIGURE 1:**
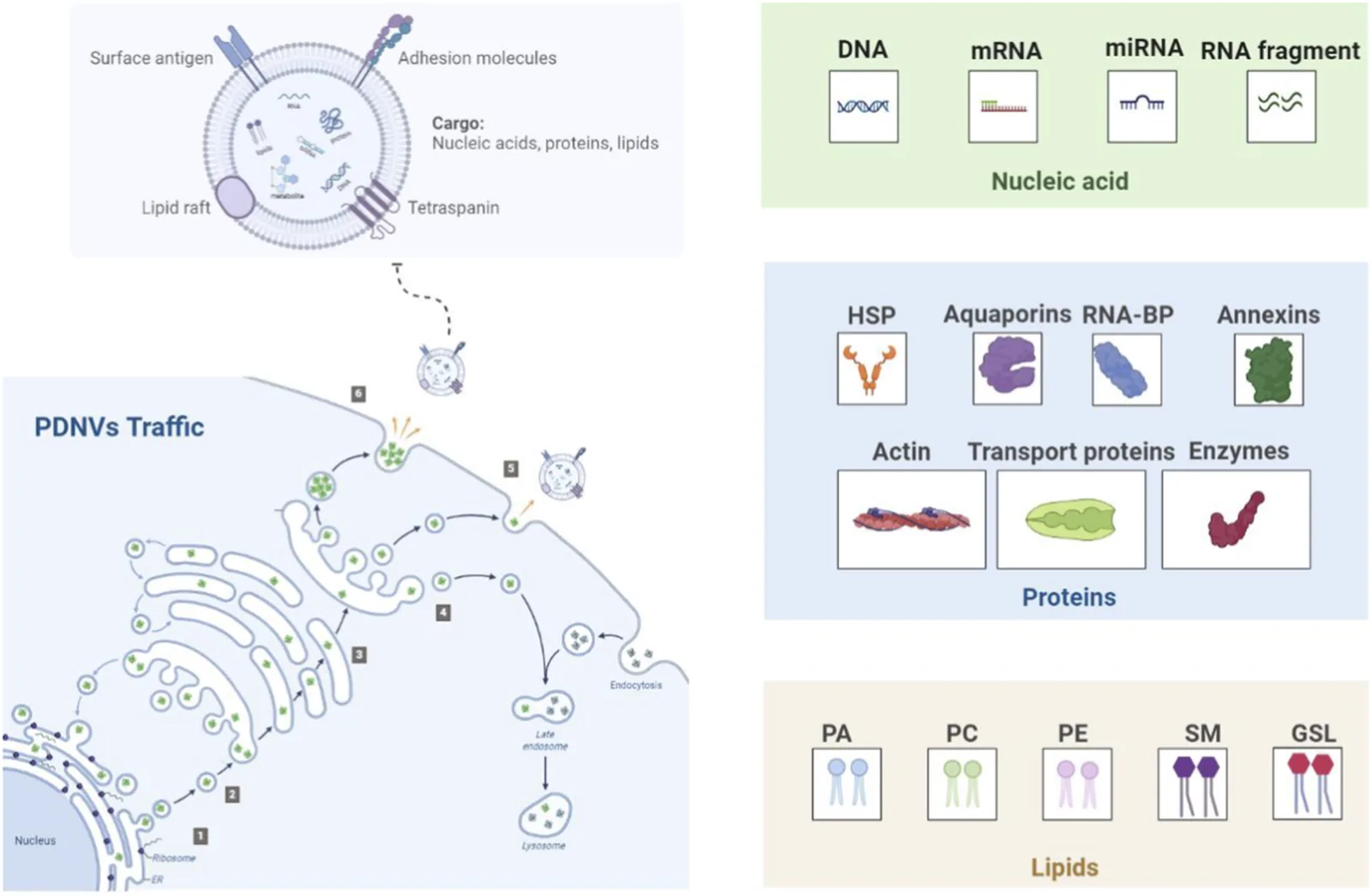
Schematic representation of the biogenesis and contents of PDNVs. Created by BioRender.

Another pathway potentially involved is the vacuolar pathway, which is closely associated with induced disease resistance and defense responses, wherein vacuoles secrete hydrolases and antimicrobial compounds into the extracellular space to counteract pathogen invasion (He et al., 2021). Plant vacuoles are multifunctional organelles involved in storage, degradation, and the maintenance of cellular homeostasis. Small vacuoles (SVs) are formed through the fusion of MVBs. These SVs subsequently undergo maturation and fusion to develop into the large central vacuole. Specifically, studies suggest that vacuole biogenesis involves the differentiation of MVBs into SVs, followed by the fusion of these SVs to generate the central vacuole. Given the role of vacuolar trafficking in secreting defense-related cargo, it is hypothesized that this pathway may also contribute to PDNV biogenesis. However, the precise mechanisms underlying this potential contribution remain to be fully elucidated (Nemati et al., 2022) (Figure 1).

### 2.3 Contents of PDNVs

The diverse biological functions of PDNVs are largely attributed to their complex and heterogeneous cargo, which primarily includes proteins, lipids, nucleic acids, and plant-specific secondary metabolites.

#### 2.3.1 Proteins

Research on the proteome of PDNVs remains less comprehensive compared to the well-characterized profiles of mammalian-derived extracellular vesicles (MDEs) (Tiwari et al., 2019). However, emerging studies highlight significant functional diversity in PDE proteins. These proteins can be broadly classified into two categories. The first category is transmembrane proteins (e.g., water channel proteins, ATPases, and ABC transporters). The second category is plastid-associated proteins. For instance, ginger-derived nanovesicles (GDNVs) contain aquaporins that enhance membrane stability (Wang X. et al., 2024), while citrus-derived vesicles comprise 600–800 proteins involved in vesicular transport (Stanly et al., 2019), including metabolic enzymes, cytokines, cytoskeletal components, and ubiquitin. Similarly, grape-derived nanovesicles include 96 plant proteins, encompassing enzymes, GTPases, and factors MVB biogenesis (Teng et al., 2022). Despite these insights, the absence of conserved, PDE-specific marker proteins poses a challenge, necessitating systematic comparative proteomics to elucidate functional mechanisms and advance translational applications.

Plant-derived nanovesicles (PDNVs) exhibit high heterogeneity. Consequently, their protein composition varies significantly based on plant species and processing conditions. Despite this variability, PDNVs commonly contain proteins associated with extracellular matrix (ECM) repair. They also contain proteins involved in antioxidant defense and stress response. The heat shock protein (HSP) family is prevalent across various plants (Martínez-Ballesta et al., 2019; Bokka et al., 2020; Pinedo et al., 2021b; Zeng et al., 2021), and functions in delaying aging by modulating cellular stress responses and promoting tissue regeneration. Further investigation into these protein mechanisms will unveil novel therapeutic targets for anti-aging and tissue repair strategies. Future research should use high-throughput proteomics combined with Western blot validation to better characterize the PDNVs proteome and explore the functional mechanisms of specific proteins in skincare applications beyond anti-aging, such as barrier repair and hydration.

#### 2.3.2 Lipids

PDNVs exhibit a complex lipid profile. This profile is integral to their membrane architecture and biological functions. The lipid composition is primarily composed of phospholipids. These include phosphatidylcholine (PC), phosphatidylethanolamine (PE), and phosphatidic acid (PA) (Cong et al., 2022; Yi et al., 2023). Additionally, sphingolipids are present,. The lipid composition differs significantly based on the plant origin (Teng et al., 2018; Yamasaki et al., 2020). These variations influence functional outcomes. For example, oral administration of ginger PDNVs mitigates high-fat diet-induced insulin resistance. This effect is achieved by modulating the Foxa2 signaling pathway. Notably, PA is identified as a key regulator through surface plasmon resonance (SPR) studies. In contrast, citrus and grape PDNVs are enriched in PC and PE. They demonstrate organ-specific targeting capabilities. For instance, they show affinity for the liver and gastrointestinal tract (Huang et al., 2023). Furthermore, garlic PDNVs include dodecanoylphosphatidylcholine (DLPC), which suppresses NLRP3 activity, offering therapeutic potential for chronic inflammatory conditions (Wang et al., 2021). Additionally, PA in ginger PDNVs interacts with bacterial proteins like HBP35, reducing pathogenicity in chronic periodontitis (Sundaram et al., 2020). Beyond these, lipids in PDNVs are organized into bilayer structures. These structures have diverse compositions. The compositions encompass glycerophospholipids and sphingolipids. Glycerophospholipids include phosphatidylinositol (PI) and phosphatidylserine (PS). Sphingolipids include ceramide (Cer), hexosylceramide (HexCer), and sphingomyelin (SM). Glycerolipids include diacylglycerol (DG), triacylglycerol (TG), and digalactosyldiacylglycerol (DGDG). Other species like cardiolipin and sterols are also present (Keshtkar et al., 2021). These lipids are essential for maintaining vesicle stability, integrity, and flexibility, enabling membrane curvature and cellular interactions. They also facilitate the crossing of physiological barriers, enhance the encapsulation of both hydrophilic and hydrophobic drugs, and improve bioavailability (Zhao et al., 2024). Techniques such as differential centrifugation and ultrasonication allow the reconstruction of these lipids into lipid-based nanovesicles for advanced drug delivery systems. Moreover, specific lipids possess inherent bioactivities (Mori et al., 2023), including anti-inflammatory properties and modulation of gut microbiota (Sundaram et al., 2020; Yamasaki et al., 2020). While the role of PDNVs lipids in anti-aging processes requires further exploration, their promise in therapeutic applications and drug delivery merits in-depth future research.

#### 2.3.3 Nucleic acids

PDNVs share similarities with mammalian EVs. They all contain proteins, lipids, and nucleic acids. However, PDNVs are characterized by a higher nucleic acid content relative to proteins. Specifically, microRNAs (miRNAs) constitute the predominant fraction (Cong et al., 2019; Teng et al., 2021; Choi et al., 2022; Qin et al., 2022; Hao et al., 2024; Shen et al., 2024). Studies analyzing PDNVs from 11 distinct plant sources have identified 418 unique miRNAs, many of which can modulate mammalian target genes, highlighting their potential functional significance (Xiao et al., 2018). For example, *ginseng*-derived nanovesicles deliver substantial miRNA cargo into bone marrow mesenchymal stem cells (BMSCs) (Xu et al., 2021), influencing genes associated with neurodifferentiation, while *ginger*-derived nanovesicles contain miR-7267–3p, which enhances IL-22 expression to alleviate intestinal inflammation (Yamasaki et al., 2020). Additionally, *konjac*-derived nanovesicles demonstrate neuroprotective properties through differentially expressed miRNAs.

Despite these findings, significant challenges persist. These challenges include batch-to-batch variability in miRNA profiles. They also include uncertainties regarding the reliability and efficacy of newly identified miRNAs. Additionally, there is an absence of comprehensive miRNA databases for many PDNVs. This lack hampers accurate miRNA annotation, discovery of novel miRNAs, and cross-species comparative analyses. Consequently, establishing standardized miRNA databases and elucidating miRNA sorting mechanisms are critical for advancing PDNVs applications. PDNVs serve as natural RNA delivery vehicles, with nucleic acid compositions—encompassing mRNAs, miRNAs, and siRNAs—determinable via agarose gel electrophoresis and varying considerably across plant species. For instance, *soybean*-derived nanovesicles exhibit high miRNA diversity (Zhang C. et al., 2016), whereas *ginger* shows lower counts; *Arabidopsis, radish*, and *Oryza sativa*-derived nanovesicles are enriched in siRNAs, and *grape*-derived nanovesicles have reduced RNA concentrations per particle (Mu et al., 2014; Zhang C. et al., 2016). Functionally, PDNVs miRNAs regulate mammalian cellular processes: *ginseng*-derived nanovesicles activate PI3K and Ras/ERK signaling to promote neural differentiation (Farberov et al., 2023), *bitter gourd*-derived nanovesicles suppress NLRP3 inflammasome activation and induce apoptosis in oral cancer cells (Cong et al., 2022), and *ginger*-derived nanovesicles target bacterial genes like *ycnE* gene to boost IL-22 production for colitis therapy (Yamasaki et al., 2020). The mechanistic action involves miRNA binding to complementary sequences in the 3′UTRs of target mRNAs, leading to translational repression or degradation (Farberov et al., 2023). Current research predominantly focuses on miRNAs, but the full scope of RNA diversity—including non-coding RNAs—remains underexplored, with limited mechanistic insights due to inadequate validation and a paucity of species-specific reference databases. Systematically characterizing the entire RNA cargo of PDNVs is essential to unlock their therapeutic and biotechnological potential.

#### 2.3.4 Secondary metabolites

PDNVs encapsulate a diverse array of small-molecule bioactive compounds and secondary metabolites, including flavonoids, saponins, polysaccharides, alkaloids, and other phytochemicals, which are intrinsically synthesized during vesicle biogenesis rather than acquired through external infiltration. These metabolites exhibit enhanced stability within vesicles compared to their free forms in source plants, positioning them as primary mediators of biological activity. In biomedical contexts, PDNVs-borne metabolites significantly influence cellular uptake and intracellular accumulation in recipient cells; for instance, β-glucan from oat-derived nanovesicles promotes dose-dependent uptake in microglia. Therapeutically, PDNV cargo is highly promising. For instance, Camellia sinensis PDNVs, abundant in flavonoids and polyphenols, provide a basis for oncological interventions, while Fragaria × ananassa PDNVs, enriched with anthocyanins and vitamins, protect MSCs from oxidative stress (Perut et al., 2021). However, industrial standardization faces challenges due to methodological inconsistencies in extraction, as evidenced by variable concentrations of active compounds like 6-gingerol and 6-shogaol in ginger PDEs isolated via different sucrose gradient protocols.

Notably, PDNVs carry plant-specific secondary metabolites, such as *aloe* emodin and aloesin in *Aloe vera*, rutin in *Sophora japonica*, and ginsenosides in *Panax ginseng*, which serve as core determinants of bioactivity (Zhang M. et al., 2016; Cao et al., 2019; Chen J. et al., 2023). For example, oat PDNVs utilize β-glucan to activate immune responses via Dectin-1 modulation (Xu et al., 2022), alleviating alcohol-induced neuroinflammation and cognitive deficits, while ginger PDNVs deliver gingerol to the liver, reducing malondialdehyde (MDA) levels and repairing ethanol-induced hepatic damage (Zhuang et al., 2015). Given this rich metabolite diversity, a pharmacology-driven approach is proposed to elucidate PDNVs’ mechanisms, leveraging their source-dependent phytochemical profiles for targeted therapeutic applications.

## 3 Bioactivity of PDNVS in skincare

The burgeoning field of PDNV research has yielded a plethora of data across various experimental models. To synthesize these findings and clarify the current state of translational readiness, Table 2​ provides a comprehensive overview of representative studies. Crucially, the evidence is stratified by experimental model to distinguish between *in vitro*, *ex vivo*, *in vivo*, and the currently limited clinical data. This stratification highlights that while mechanistic insights are robust at the cellular level, clinical validation remains an emergent Frontier.

**TABLE 2:** Summary of verified plant-derived nanovesicles bioactivity evidence in skincare.

Plant species	Bioactivity reported	Key bioactive components	Mechanism/Pathway	Model system	Evidence level	Ref
*Aloe vera*	Photoaging protection, antioxidant, anti-aging	Flavonoids, phenolic acids, polysaccharides	Nrf2/ARE activation (↑HO-1, NQO-1, SOD), ↓MDA, ↓β-gal/SASP	HaCaT, HDF (UVB/UVA), ICR mice (microneedling-assisted)	*In vitro* + *In vivo*	(Sun et al., 2025c);
*Polygonum multiflorum*	Photoaging protection, collagen synthesis	Anthraquinones, flavonoids, phenolic acids	Nrf2 activation, ↓MMP-1, ↑COL1/COL3	HDF (UVB), nude mouse photoaging model	*In vitro* + *In vivo*	(He et al., 2024)
*Vitis vinifera*	Photoaging mitigation, antioxidant effects	Resveratrol, polyphenols	ROS scavenging, ↓epidermal thickness, attenuated collagen loss, ↑cutaneous antioxidant capacity; single-cell RNA sequencing confirms epithelial proliferation/differentiation protection	HaCaT (UVB), kM mouse photoaging model	*In vitro* + *In vivo*	(Wang et al., 2025b)
*Panax ginseng*	UV protection, antioxidant, anti-aging	Ginsenosides (Re, Rg1, Rb1)	AP-1 signaling suppression, ↓MMP-2/3, ↓COX-2, ↓IL-6, ↓p21	HaCaT (UVB/H_2_O_2_)	*In vitro*	(Choi et al., 2024)
*Citrus × paradisi*	Wound healing, antioxidant, anti-inflammatory	Naringin, hesperidin, PC/PE lipids	↓TNF-α/IL-6, ROS scavenging, promoted HUVEC tube formation	HaCaT, mouse excisional wound model	*In vitro* + *In vivo*	(Wang et al., 2014)
*Fragaria × ananassa*	Antioxidant, cytoprotection	Anthocyanins, vitamin C, folic acid, flavonoids	ROS scavenging, Nrf2 activation	Human mesenchymal stem cells (MSCs)	*In vitro*	(Perut et al., 2021)
*Allium sativum*/*Allium cepa* var. *aggregatum*	Psoriasis treatment	Not fully characterized	↑NRF2, ↓IL-17/NF-κB, ↑KRT10/IVL	HaCaT, IMQ-induced psoriasis mouse model, porcine skin explants	*In vitro* + *In vivo* + *Ex vivo*	(Kalarikkal et al., 2025)
*Portulaca oleracea*	Atopic dermatitis relief	Polysaccharides (POP), proteins	M1→M2 macrophage polarization, ↓NF-κB/STING signaling	RAW264.7, DNCB-induced atopic dermatitis mouse model (microneedling-assisted)	*In vitro* + *In vivo*	(Long et al., 2025)
*Curcuma amada*	Diabetic wound healing	Curcuminoids, gingerols	↓KSRP, ↑FSTL1, promoted HaCaT migration	HaCaT, STZ-induced diabetic mouse wound model, porcine skin explants	*In vitro* + *In vivo* + *Ex vivo*	(Suresh et al., 2025);
*Dendropanax morbifera*	Whitening, anti-melanogenic	Not fully characterized	↓MITF/TYR/TRP-1/2, inhibited α-MSH-MC1R-cAMP axis	B16F10 melanoma cells, full-thickness human epidermal model	*In vitro*	(Lee et al., 2020a)
*Pachyrhizus erosus*	Anti-melanogenic, wound repair	Not fully characterized	↑Collagen expression, ↓melanocyte activity	HDF, zebrafish larvae	*In vitro* + *In vivo*	(Kusnandar et al., 2025b)
*Rosa* sp.	Whitening, photoaging relief†	Not fully characterized	Reduced melanin synthesis (microneedling-assisted)	Human clinical observation (*n* = 12)	Human (clinical)	(Kleebayoon and Wiwanitkit, 2024; Theodorakopoulou et al., 2024)

† Microneedling-assisted delivery; clinical observation sourced from commercial reports, not peer-reviewed.

### 3.1 Antioxidant and anti-aging effects

Skin aging is one of the most evident signs of human senescence. Its mechanisms encompass a complex and diverse interplay. This interplay includes biochemical reactions, genetic programming, and external stimuli. Among these, alterations in oxidative balance emerge as a primary cellular disturbance contributing to skin aging. Reactive oxygen species (ROS) are continuously generated through cellular oxidative metabolism during chronic aging processes, while environmental factors such as ultraviolet radiation, pollution, smoking, and inadequate nutrition further exacerbate the imbalance in oxidative balance. As aging progresses, the skin gradually loses its natural functional properties and regenerative capacity, an increased incidence of autoimmune and neoplastic diseases (Papaccio et al., 2022). Therefore, anti-aging and antioxidant strategies are crucial for maintaining human health.

PDNVs isolated from *Malus domestica* fruits, which enhance the expression of type I collagen alpha 1 (COL1A1) and fibrillin 1 (FBN1) in dermal fibroblasts (Seo et al., 2021). Similarly, nanovesicles derived from *Centella asiatica* callus tissue, achieved through illumination with magenta LED light, results in a more significant enhancement of the expression of key skin health-related proteins, including filaggrin (FLG), aquaporin 3 (AQP3), and collagen type I (COL1) (Kim M.-J. et al., 2023). This finding further confirms the positive role of PDNVs in skin anti-aging. Potato-derived nanovesicles exhibit unique penetration capabilities, which not only promote the proliferation of HaCaT cells but also prevent collagen degradation by inhibiting the expression of collagenolytic enzymes MMP-1, MMP-2, and MMP-9, as well as inflammatory cytokines IL-6 and TNF-α. Furthermore, these nanovesicles induce the expression of cellular detoxifying enzymes, such as glutathione S-transferase alpha 4, thereby protecting and ameliorating photodamage in HaCaT cells (Lee et al., 2023a).

Ginseng-derived nanovesicles (GrDNVs) significantly enhance the expression of High Mobility Group Box 1 (HMGB1) in a dose-dependent manner, effectively inhibiting UVB-induced apoptosis. They also exhibit notable anti-pigmentation effects by precisely modulating the AP-1 signaling pathway, which suppresses the expression of MMPs, COX-2, and IL-6 (Couch et al., 2024). Additionally, GrEVs, along with *iris*-derived nanovesicles, are capable of regulating the p38/JNK MAPK pathway, reducing the levels of p53 and p21, thereby effectively inhibiting cellular senescence and subsequently enhancing skin health (Cho et al., 2021; Kim J.-S. et al., 2023). Comparative analyses revealed that ginseng-derived nanovesicles outperformed raw plant extracts in reducing the expression of aging biomarkers (MMP12, MMP13, and NOTCH3). Similarly, *Camellia sinensis* (green tea)-derived nanovesicles downregulated MMP13 and NOTCH3. Notably, PDNVs treatment uniquely upregulated fibroblast growth factor 12 (FGF12), a key mediator of skin regeneration. Furthermore, vimentin (VIM)—a marker of mesenchymal cells involved in wound contraction—exhibited an upward trend exclusively in the PDNV-treated groups, suggesting enhanced dermal structural support (Cho et al., 2022).

*Dendrobium*-derived nanovesicles (DDNVs) exhibited a remarkable capacity to accelerate skin wound healing in C57 mice. *In vivo* experiments confirmed that DDNVs intervention effectively suppressed the expression of interleukin-1β (IL-1β), thereby accelerating the repair of skin wound tissue. These findings underscore the efficacy of DDNVs in both suppressing IL-1β expression and enhancing wound healing processes (Tu et al., 2024). Opuntia ficus-indica-derived nanovesicles (FicoVes) demonstrated a dose-dependent acceleration of epithelial wound closure in L929 fibroblast monolayers, thereby underscoring their promising potential in facilitating tissue repair processes (Naselli et al., 2024).

Nanovesicles from *C. asiatica* callus cells exhibit a concentration-dependent ability to inhibit the production of reactive oxygen species (ROS) (Logozzi et al., 2021). The grape and tomato-derived nanovesicles (GT-HNVs) exhibited remarkable stability, enhanced radical-scavenging abilities, and superior cellular uptake efficiency. Notably, they significantly decreased the levels of reactive oxygen species (ROS) and elevated the activities of antioxidative enzymes in L-02 cells (Wang J. et al., 2024).

The nanovesicles derived from *iris* and *Aloe vera* upregulate the transcription levels of antioxidant genes, including heme oxygenase-1 (HO-1), catalase, and superoxide dismutase (SOD), through the activation of the Nrf2 gene. This process subsequently enhances the antioxidant defense mechanism, alleviates oxidative stress responses, and facilitates wound healing and normal differentiation of skin cells (Kim et al., 2021). The vesicles derived from strawberry and lemon exhibit a robust antioxidant effect, protecting human mesenchymal cells from oxidative stress (Logozzi et al., 2021).

### 3.2 Whitening effects

The synthesis of melanin is an exceptionally complex process. It primarily involves three key enzymes. These enzymes belong to the tyrosinase gene family. They are Tyrosinase (TYR), Tyrosinase-related protein-1 (TRP-1), and Tyrosinase-related protein-2 (TRP-2). Given that dysregulated melanogenesis underlies hyperpigmentation disorders and melanoma, targeting the melanin synthesis pathway is crucial. Melanoma incidence continues to rise globally, often exhibiting resistance to conventional therapies, underscoring the need for novel inhibitory agents (Sung et al., 2021).

In a study, researchers compared the nanovesicles derived from the stems of *Dendropanax morbifera* (DMNVs) with those derived from its leaves. They found that DMNVs exhibited more prominent effects. Specifically, DMNVs inhibited melanogenesis. Specifically, DMNVs were able to suppress the expression of MITF in the α-MSH-MC1R pathway in melanoma cells, further inhibiting the expression of TYR, TRP-1, and TRP-2. The inhibitory effect of DMNVs was even superior to that of commonly used arbutin, and neither showed significant cytotoxicity. This discovery suggests that DMNVs, as a novel natural candidate, hold great potential as an anti-melanogenic agent in pharmaceutical and cosmetic formulations (Lee et al., 2020a).

*Pachyrhizus erosus*-derived nanovesicles (PEDNVs) demonstrated potential anti-melanogenic effects in zebrafish experiments. The study further revealed that PEDNVs, at a concentration of 2.5 μg/mL, could promote the development of the extracellular matrix, aiding in wound repair processes associated with skin regeneration (Kusnandar et al., 2025a). Although various melanin inhibitors are currently used in cosmetics, many of them have side effects. In contrast, plant-derived nanovesicles exhibit significant advantages in the development of skin whitening agents due to their non-toxic and side-effect-free characteristics.

### 3.3 Anti-inflammatory and regenerative effects

**FIGURE 2:**
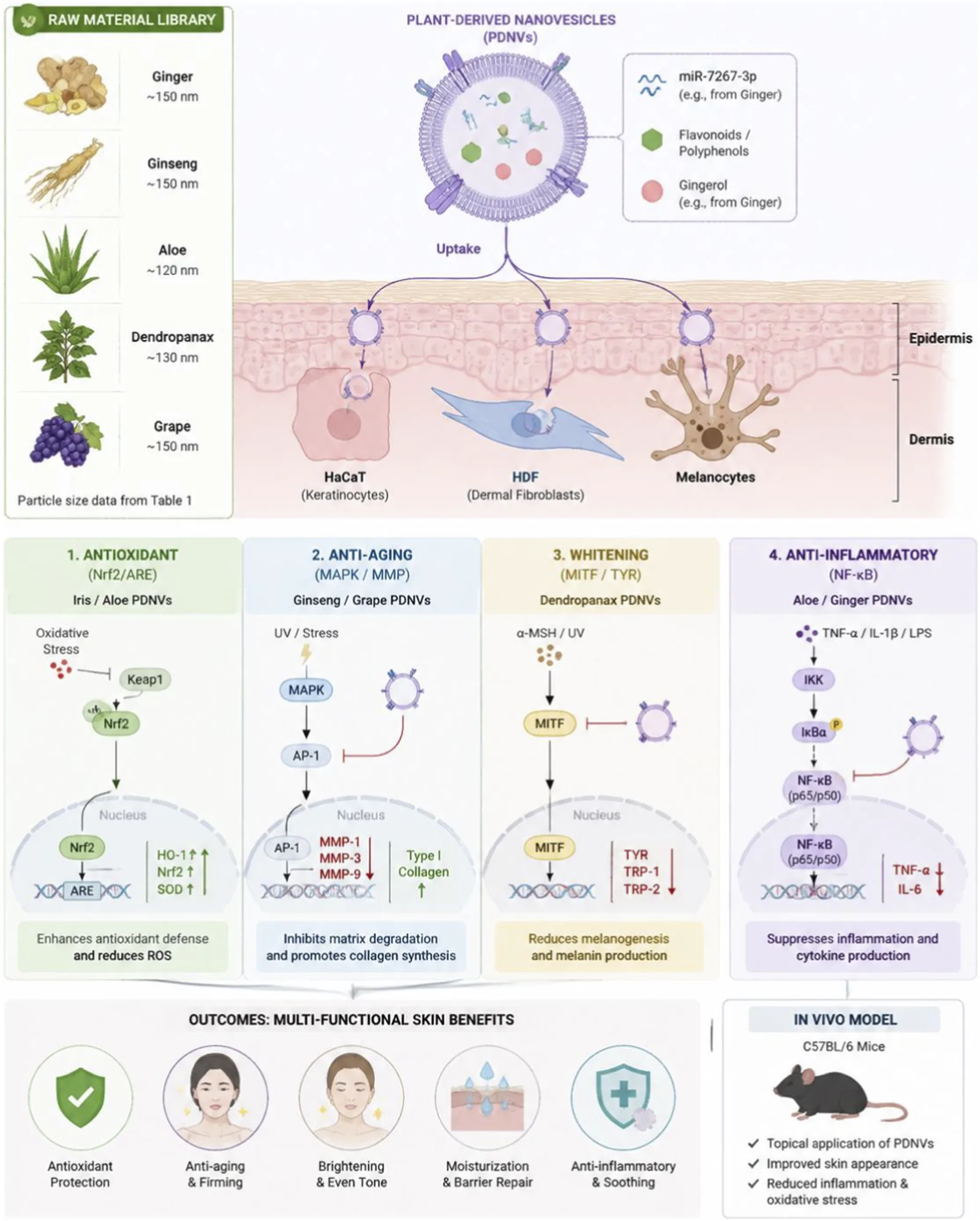
Bioactivity of PDNVs in skincare. Created by BioRender.

*Ginseng*-derived nanovesicles (GDNVs), *Aloe vera* peel-derived nanovesicles, and *wheat*-derived nanovesicles all effectively augment proliferation and migration capabilities of skin cells, including HaCaT keratinocytes, HDF fibroblasts, and HUVEC endothelial cells. These vesicles play a crucial role in the vascularization phase of wound healing. They exert a positive influence on skin wound repair. GDNVs not only mitigate wound inflammatory factors but also regulate skin cell cycles, activate the TGF-β pathway, thereby strengthening cytoskeletal structures, and significantly promote skin cell proliferation. This offers a novel solution for wound healing (Yang et al., 2023a) Studies have shown that GDNPs further enhance skin wound healing and reduce inflammation by modulating the ERK and AKT/mTOR pathways (Kim and Park, 2022). *Aloe vera*-derived nanovesicles (AVDNVs) are enriched with flavonoids and phenolic acids, contributing to their potent antioxidant and anti-inflammatory properties. Studies demonstrate that AV-PDNVs significantly decrease the secretion of pro-inflammatory cytokines, including TNF-α, IL-6, and IL-1β exhibiting exceptional inflammatory regulatory capabilities. These findings suggest their potential to reduce scar formation at the cellular and molecular levels during the early stages of wound healing (Huang et al., 2024). Research has shown that nanovesicle derived from *Tomato* (Savcı et al., 2023), *Pomegranate* (Sánchez-López et al., 2019), and *Dendrobium* all exhibit anti-inflammatory effects that are beneficial for chronic wound healing. *Tomato*-derived nanovesicles induce the migration of human keratinocytes and mouse fibroblasts, thereby promoting wound healing. *Dendrobium*-derived nanovesicles can inhibit the expression of IL-1β in mouse wounds, exhibiting anti-inflammatory effects and promoting wound healing. Complementing these findings, PDNVs often encapsulate broad-spectrum antimicrobial phytochemicals. For instance, the terpene-rich profile characteristic of *Lavandula officinalis*-derived nanovesicles underpins their potential to combat skin pathogens (Haddou et al., 2023; Meziane et al., 2025). Bioactivity of PDNVs in skincare is illustrated in Figure 2.

### 3.4 Integrated mechanistic pathways of PDNVs in skin biology

The dermatological efficacy of plant-derived nanovesicles (PDNVs) is orchestrated through a hierarchically organized network of signaling cascades that converge on core skin physiology nodes, with each pathway validated by source-specific, model-matched original studies.

The Nrf2/ARE antioxidant axis​ serves as the primary defense node: *Aloe vera*-derived nanovesicles promote Nrf2 nuclear translocation in UVB-irradiated HaCaT keratinocytes and SKH-1 hairless mouse skin, upregulating cytoprotective enzymes including HO-1, NQO1, SOD, and CAT while reducing malondialdehyde (MDA) accumulation to delay photoaging phenotypes (Sun et al., 2025c); *Polygonum multiflorum*-derived nanovesicles exert similar Nrf2-activating effects in UVB-exposed HaCaT cells and nude mouse skin, concurrently suppressing MMP production and elevating type I/III collagen expression (He et al., 2024); garlic- and shallot-derived nanovesicles further engage this axis in imiquimod (IMQ)-induced psoriasis mouse models and porcine skin explants, coupling Nrf2 activation with IL-17 suppression via direct crosstalk with the NF-κB pathway (Kalarikkal et al., 2025). Parallel to this antioxidant network, NF-κB-mediated inflammatory cytokine regulation​ defines the immune-modulatory node: *Aloe vera* peel-derived nanovesicles inhibit IKK-mediated IκB phosphorylation in LPS-stimulated RAW264.7 macrophages and HaCaT keratinocytes, blocking p65 nuclear translocation to suppress pro-inflammatory cytokines (TNF-α, IL-1β, IL-6, IL-17) while sparing anti-inflammatory IL-10 and TGF-β (Ramírez et al., 2024); *Portulaca oleracea*-derived nanovesicles delivered via microneedles drive M1-to-M2 macrophage phenotypic switching in DNCB-induced atopic dermatitis murine models by suppressing both NF-κB and STING signaling, reducing epidermal thickening and pruritus (Long et al., 2025); *Dendrobium*-derived nanovesicles similarly suppress IL-1β expression in C57BL/6 mouse wound tissue to accelerate re-epithelialization (Tu et al., 2025).

The MAPK/p38/JNK-AP-1/MMP catabolic axis​ governs photoaging-related extracellular matrix (ECM) degradation: UVB-induced ROS activate p38, JNK, and ERK MAPKs, which drive AP-1 transcriptional activation and the subsequent upregulation of MMP-1, MMP-2, MMP-3, and MMP-9 that cleave dermal collagen; ginseng root-derived nanovesicles blunt this cascade in UVB-irradiated HaCaT cells, downregulating AP-1 target genes including MMP-2, MMP-3, COX-2, and IL-6 (Choi et al., 2024); potato-derived nanovesicles exert analogous MAPK-inhibitory effects in H_2_O_2_-treated HaCaT cells, reducing MMP-1/2/9 expression alongside IL-6 and TNF-α while inducing detoxification enzyme GST-α4 (Lee et al., 2023b); notably, Kyoho grape (Vitis vinifera)-derived nanovesicles ameliorate UV-induced photoaging in both HaCaT keratinocytes and kM mouse models by reducing epidermal thickness, minimizing collagen fiber loss, and enhancing cutaneous antioxidant capacity, with single-cell RNA sequencing further revealing that nanovesicles protect epithelial cells by modulating their proliferation and differentiation trajectories (Wang M. et al., 2025).

Counterbalancing this catabolic axis, the TGF-β/Smad anabolic pathway​ drives collagen synthesis: *Polygonum multiflorum*-derived nanovesicles promote Smad2/3 phosphorylation and nuclear translocation in UVB-exposed models, upregulating COL1A1 and COL3A1 transcription while inhibiting MMP-1 (Park et al., 2025); *Aloe vera*-derived nanovesicles further suppress TGF-β1-induced myofibroblast differentiation and pathological collagen contraction *in vitro* (Ramírez et al., 2024); ginseng-derived nanovesicles activate TGF-β signaling in human dermal fibroblasts (HDFs) to strengthen cytoskeletal organization and promote proliferation (Yang et al., 2023b).

The ERK/AKT/mTOR proliferative axis​ supports wound healing and tissue repair: *Curcuma amada* (mango ginger)-derived nanovesicles enhance HaCaT migration and re-epithelialization in streptozotocin (STZ)-induced diabetic mouse wounds and porcine skin explants via KSRP-mediated upregulation of follistatin-like 1 (FSTL1), a novel pro-reparative node within this pathway (Suresh et al., 2025); mint leaf-derived nanovesicles accelerate infected wound closure in rat models by modulating bacterial membrane elasticity and dampening local inflammation, coupling ERK/AKT activation with enhanced keratinocyte proliferation (Saroj et al., 2025).

At the pigmentary regulation interface, the MITF/TYR/TRP melanogenesis axis​ controls hyperpigmentation: α-MSH binding to the MC1R receptor activates cAMP-PKA signaling to upregulate microphthalmia-associated transcription factor (MITF), which drives transcription of tyrosinase (TYR) and tyrosinase-related proteins (TRP-1/TRP-2); *Dendropanax morbifera* leaf-derived nanovesicles outperform arbutin in B16F10 melanoma cells and a full-thickness human epidermal model, suppressing MITF, TYR, TRP-1, and TRP-2 expression via inhibition of the α-MSH-MC1R-cAMP axis without cytotoxicity (Lee et al., 2020b); *Pachyrhizus erosus*-derived nanovesicles exert analogous MITF-inhibitory effects in HDFs and zebrafish larvae, concurrently promoting ECM collagen expression to support post-inflammatory repigmentation repair (Kusnandar et al., 2025b). Critically, these pathways do not operate in isolation but engage in hierarchical molecular crosstalk: Nrf2 activation antagonizes NF-κB signaling by reducing ROS-mediated IKK activation, explaining why PDNVs with potent Nrf2-activating cargo (e.g., Aloe vera, garlic) concomitantly dampen oxidative stress and inflammation; the MAPK/AP-1/MMP and TGF-β/Smad axes jointly determine net ECM turnover, such that *P. multiflorum* nanovesicles shift the balance toward collagen preservation by simultaneously suppressing catabolic enzymes and stimulating anabolic synthesis; ERK/AKT signaling further cooperates with TGF-β/Smad during re-epithelialization, coordinating keratinocyte proliferation with matrix deposition. This multi-node molecular engagement constitutes the mechanistic basis for the broad, pleiotropic bioactivity of PDNVs in dermatology, though current mechanistic insights remain largely derived from *in vitro* inference with a pressing need for *in vivo* single-cell omics and live-cell imaging to deconvolute cargo-specific pathway activation for rational PDNVs therapeutic design (Table 2).

### 3.5 Cross-cutting evidence quality assessment

To provide a systematic overview of the evidence supporting PDNV bioactivity claims in skincare, this section summarizes the available evidence by claim type and model system, accompanied by a GRADE-informed quality assessment. This cross-cutting analysis enables direct comparison of evidence strength across different claim categories, highlighting the translational gaps between preclinical observations and clinical applicability (Table 3).

Current PDNVs research exhibits a “preclinical richness but clinical poverty” paradox: while *in vitro* mechanistic insights are robust, translational progression is hampered by a lack of standardized pharmacokinetic data and rigorous human trials. Anti-aging and anti-inflammatory claims, though supported by moderate animal evidence, lack validation via randomized controlled trials (RCTs). Whitening claims remain speculative due to reliance on non-mammalian models and assisted-delivery clinical observations. Future investigations must prioritize translational pipelines—including Franz cell diffusion studies and RCTs—to substantiate preclinical promises.

**TABLE 3:** Cross-cutting evidence quality assessment of PDNV bioactivity claims in skincare.

Claim type	*In vitro* evidence	Animal model evidence	Human/Clinical evidence	Overall GRADE*
Antioxidant/Anti-aging​	Strong: Nrf2/ARE activation, MMP inhibition, and collagen upregulation consistently replicated across ≥6 PDNV sources (Aloe vera, Polygonum multiflorum, Kyoho grape, ginseng).	Moderate: UVB mouse models show improved MDA/SOD ratios and reduced wrinkle scores, though histological validation varies across studies.	Very weak: Limited to small-sample (n = 12) microneedling-assisted observations lacking placebo control.	Low​
Anti-inflammatory​	Strong: NF-κB inhibition and pro-inflammatory cytokine reduction (TNF-α, IL-6, IL-1β) reproducible across multiple PDNV sources.	Moderate: IMQ psoriasis and DNCB atopic dermatitis models show reduced epidermal thickness and improved clinical scores.	Very weak: No standalone clinical endpoints; effects inferred from *ex vivo* porcine skin penetration data.	Low​
Whitening/Anti-melanogenic​	Moderate: MITF/TYR/TRP pathway characterization robust in B16 cells and 3D human epidermal models.	Weak: Reliance on non-mammalian zebrafish models; no validated mammalian *in vivo* whitening data available.	Very weak: Single case series involving microneedling; no evidence of passive transdermal penetration.	Very low​
Wound healing​	Strong: Enhanced proliferation and migration of HaCaT, HDF, and HUVEC cells well-documented across multiple PDNV sources.	Moderate: Improved wound closure rates in STZ-diabetic mouse and excisional wound models.	Absent: No registered clinical trials for PDNV-based wound healing interventions.	Low​

* GRADE, quality assessment: High = further research is very unlikely to change confidence; Moderate = further research may impact confidence; Low = further research is likely to change confidence; Very low = any estimate is very uncertain.

## 4 Application of PDNVS in skincare

### 4.1 Direct application

Owing to their multifaceted efficacy, including antioxidant, anti-aging, whitening, anti-inflammatory, and regenerative properties, PDNVs can be directly incorporated as active cosmetic ingredients into various formulations (Mu et al., 2023). These formulations include toners, emulsions, creams, serums, and masks, where PDNVs can exert their beneficial effects directly on the skin. The current application landscape is evidenced by a growing body of scientific literature, patents, and an increasing number of commercial brands that are exploring or have incorporated PDNVs derived from plants like ginseng, aloe vera, grapefruit, and grape into their skincare products. These products often claim specific benefits such as enhanced moisturization, soothing, anti-aging, and skin barrier repair.

### 4.2 Application as delivery systems

Beyond their intrinsic bioactivity, the inherent nanostructure and lipid bilayer composition of PDNVs make them excellent natural delivery vehicles for encapsulating and delivering both hydrophilic and hydrophobic active ingredients. PDNVs demonstrate significant advantages over conventional lipid-based or synthetic polymeric nanoparticles. These advantages encompass multiple aspects. First, they have inherent therapeutic properties due to their botanical origins and cargo composition. Second, they offer enhanced bioavailability. Cellular uptake rates are approximately double that of liposomes. This is observed in grapefruit-derived PDNVs (Stanly et al., 2019). It is potentially attributed to unique surface membrane proteins or carbohydrate molecules (Hu and Palić, 2020). Third, they provide superior drug protection capabilities. They shield encapsulated active substances from degradation in external environments (Chen X. et al., 2023). This is evidenced by detectable fluorescent signals in multiple intestinal regions post-oral administration. Additionally, they show resilience to gastric acid and digestive enzyme breakdown. This is supported by ζ-potential stability in ginger PDNVs under varying pH conditions. Fourth, they exhibit reduced immunogenicity. They do not contain zoonotic or mammalian pathogens. No reported immune stress responses occur even at high doses (e.g., 10^11^ particles or 1,000 ng/mL). High cellular uptake efficiency is maintained throughout.

Although safety concerns persist regarding compositional variations, further evidence is needed to confirm risks. PDNVs function as multifunctional delivery systems in tissue repair, exemplified by ginger PDNVs delivering siRNA to colon tissue to suppress inflammatory microenvironments and promote epithelial regeneration (Yang et al., 2022), and ginseng root PDNVs engineered with neutrophil membranes to deliver miRNA targeting the NOX4 pathway, ameliorating mitochondrial dysfunction and facilitating acute lung injury repair (Ma et al., 2021). Moreover, PDNVs serve as ideal platforms for anti-aging applications, such as combining with Nicotinamide Mononucleotide (NMN) through active loading methods to enhance stability and absorption efficiency (Wang et al., 2022), addressing NMN’s sensitivity to environmental factors like aqueous solutions and light. While plant-derived nanovesicles offer excellent biocompatibility, the field can draw inspiration from the stability and tunability of synthetic architectures (El Yousfi et al., 2023). In summary, the multifunctionality of PDNVs promises advanced applications in precision medicine and anti-aging therapies.

### 4.3 Engineering modification

To enhance the functionality, stability, or targeting specificity of PDNVs, various engineering strategies are being explored. One approach involves applying specific external stimuli during the growth of plant source materials (whole plants or callus cultures) (Meng et al., 2021). These stimuli can include specific wavelengths of light (e.g., magenta LED light used for *Centella asiatica* callus to enhance skin barrier protein expression), sound waves (music), or chemical inducers (e.g., hormones, elicitors), which can alter the plant’s metabolism and subsequently lead to the production of PDNVs with enhanced or novel bioactivities.

**FIGURE 3:**
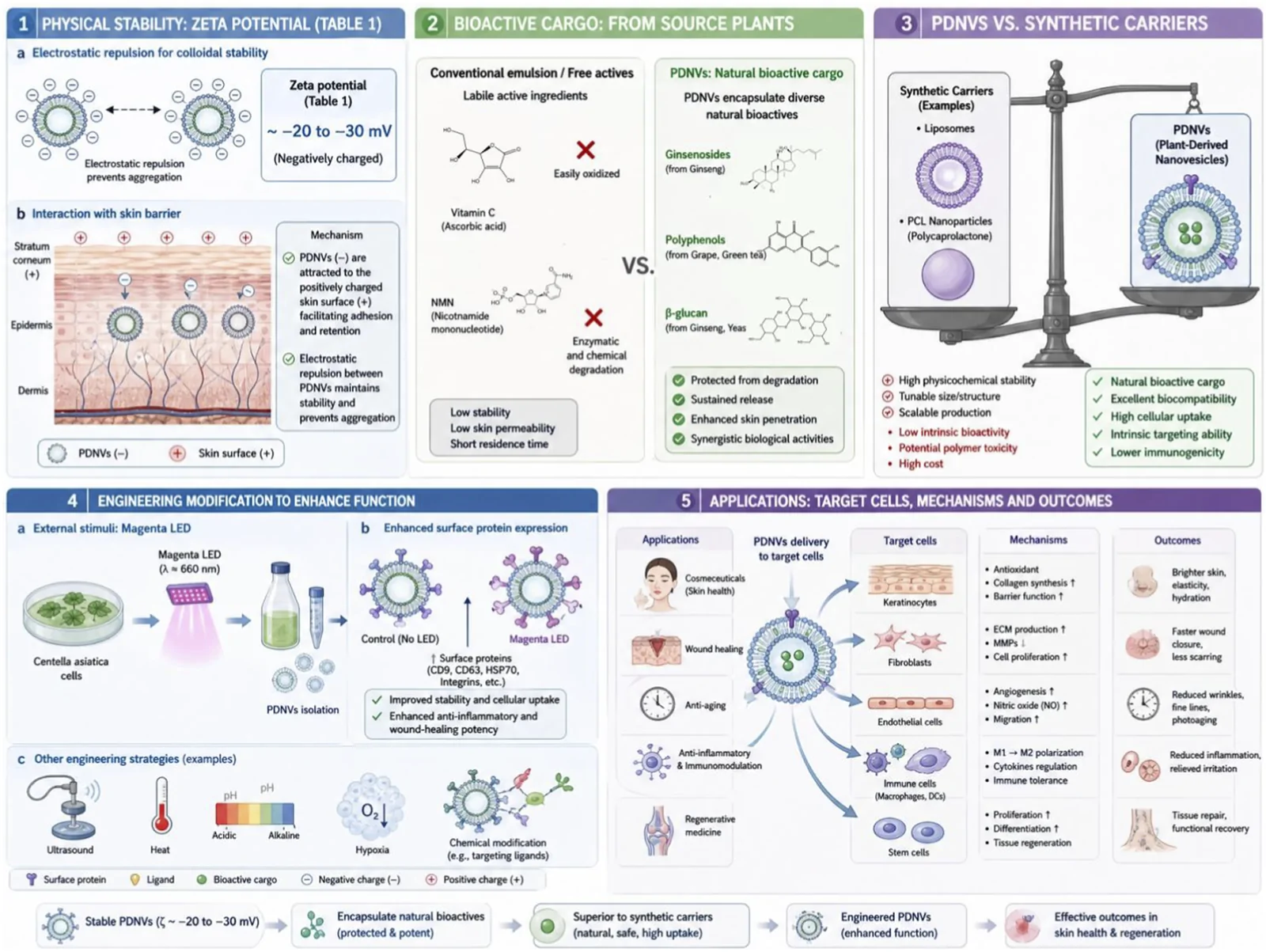
Application of PDNVs in skincare. Created by BioRender.

A more advanced and prospective strategy involves the genetic editing of plant cells themselves. Using technologies like CRISPR-Cas9, plant cells could be programmed to produce PDNVs that carry specific therapeutic proteins (Ma et al., 2024), RNAs, or have modified surface properties for improved targeting. However, the application of genetically modified plants for PDNVs production raises important safety and regulatory considerations that must be thoroughly addressed before clinical or cosmetic translation (Chen et al., 2025). Application of PDNVs in skincare is illustrated in Figure 3.

## 5 Industrial production of PDNVS

### 5.1 Extraction methods and industrial production technology

In the field of nanovesicles research, five major categories of isolation and purification approaches have been established for these nano-sized vesicles. These methodologies include ultracentrifugation, size-based separation techniques, precipitation, immunoaffinity capture, and microfluidic techniques. Here, we provide a concise summary of the principles, advantages, and limitations of these techniques, as outlined in Table 4.

**TABLE 4:** Advantages and disadvantages of different PDNVs extraction methods.

Isolation technique	Isolation principle	Advantages	Disadvantages
Polymer Precipitation	Hydrophilic polymers interact with water molecules surrounding PDNVs, reducing PDNVs solubility and inducing precipitation.	Simple operation, no requirement for high-precision equipment, compatible with large sample volumes, and high yield	Tedious sample preparation, low purity, and difficulty in achieving standardized procedures
Size-based Techniques	1. Ultrafiltration: Solute filtration is performed using membranes with specific pore sizes 2. Size-exclusion chromatography: The solution is passed through a chromatographic column, where solutes of different volumes elute at varying rates	1. Fast, convenient, portable; capable of isolating PDNVs subpopulations 2. Higher purity, minimal impact on PDNVs structure and activity, simple collection; capable of isolating PDNV subpopulations	1. Membrane clogging may occur; cannot distinguish other impurities of the same volume; may affect PDNVs structure and activity 2. Requires professional equipment; cannot distinguish other impurities of the same volume
Ultracentrifugation-based Techniques	1. Sequential ultracentrifugation: Different centrifugal forces are applied to separate solutes based on their volume, mass, and sedimentation coefficient. 2. Gradient ultracentrifugation: Solutes are separated by density under a constant centrifugal force.	1. Low cost, compatible with large sample volumes, and high yield 2. Higher purity; capable of isolating PDNV subpopulations	1. Heavy workload, time-consuming, poor portability, low purity; may damage PDNVs structure 2. Heavy workload, time-consuming, poor portability; may damage PDNVs structure and cannot distinguish other impurities with the same density
Microfluidics-based Techniques	Enables the control, manipulation, and detection of PDNVs at the microscale.	High automation, high efficiency, and excellent portability	Complex equipment, lack of large-scale clinical sample testing validation, and limited sample volume compatibility
Immunoaffinity Capture	Exogenous antibodies bind to PDNVs surface antigens through antigen-antibody immune reactions, forming immune complexes; high-purity PDNVs can be obtained after elution.	Able to isolate PDNVs from specific sources and obtain PDNV subpopulations with extremely high purity	Needs to establish improved exosomal markers; cannot identify PDNVs with different surface proteins; low yield, small sample volume compatibility, high cost; may damage PDNV structure and activity

The translation of PDNVs from laboratory research to industrial-scale applications requires the development of efficient, scalable, and cost-effective production technologies. For larger scales, Tangential Flow Filtration (TFF) is highly promising due to its scalability, continuous processing capability, and relatively gentle handling of vesicles, which helps preserve their integrity and biological activity (Luo et al., 2025). Often, a combination of techniques is employed to optimize yield and purity, such as using TFF for initial concentration and clarification followed by PEG precipitation for further purification. Emerging technologies like microfluidic-based purification and affinity chromatography are also under investigation for their potential to offer higher resolution and efficiency in PDNVs isolation (Yuan et al., 2023).

### 5.2 Quality control

Rigorous quality control is paramount to ensure the batch-to-batch consistency, safety, and efficacy of PDNVs for cosmetic applications. Key parameters that must be monitored include: (i) Physicochemical properties: Particle size distribution, particle concentration, and zeta potential (surface charge), typically characterized using techniques like Nanoparticle Tracking Analysis (NTA) or nano-flow cytometry (Kilasoniya et al., 2023). (ii) Morphology: Visual assessment of vesicle morphology using Transmission Electron Microscopy (TEM) or Atomic Force Microscopy (AFM) (Sun et al., 2025d). (iii) Compositional Fingerprinting: Profiling of molecular cargo using techniques such as High-Performance Liquid Chromatography (HPLC) for metabolites, protein electrophoresis (SDS-PAGE) for proteins, and thin-layer chromatography (TLC) for lipids. (iv) Identification of Characteristic Markers: The use of combinatorial approaches (e.g., Western blot, PCR, mass spectrometry) to identify specific marker molecules (proteins, RNAs, metabolites) is crucial for standardization and quality assessment (Liu et al., 2025).

### 5.3 Stability of PDNVs

**FIGURE 4:**
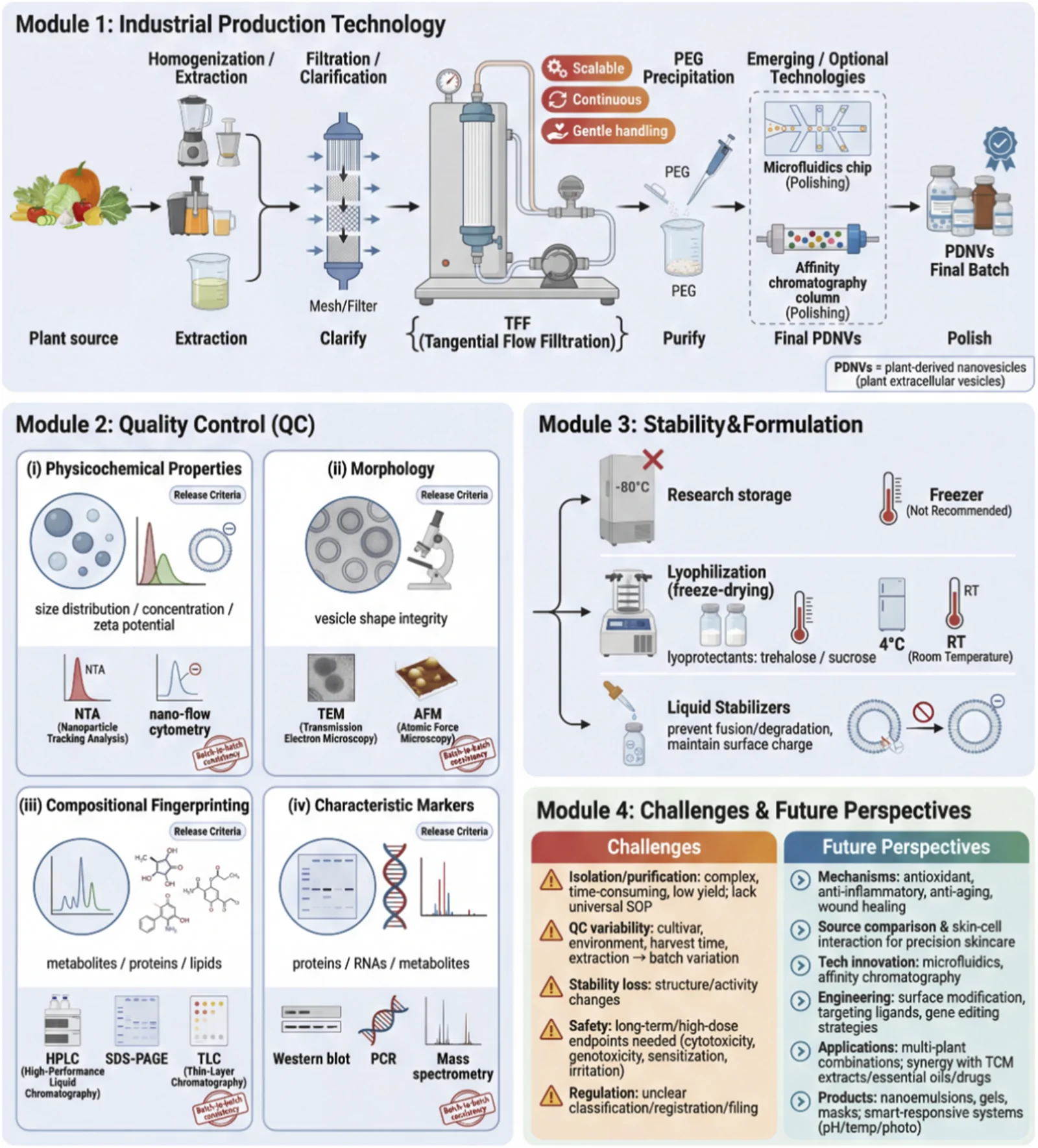
Industrial production of PDNVs. Created by BioRender.

The stability of PDNVs during storage, transportation, and incorporation into final products is a critical factor determining their shelf life and practical efficacy. While storage at −80 °C is common in research settings, it is impractical for large-scale commercial distribution and consumer use (Abraham et al., 2022). Lyophilization (freeze-drying) using lyoprotectants such as trehalose or sucrose can significantly enhance the stability of PDNVs, allowing for storage at higher temperatures (e.g., 4 °C or even room temperature) for extended periods without significant loss of integrity or activity (Noguchi et al., 2019). Concurrently, the development of liquid stabilizers that protect membrane integrity (e.g., by preventing fusion or degradation) and maintain surface charge is crucial for formulating stable liquid-based PDNV products suitable for industrial production, storage, and transport (Karn et al., 2021). Industrial production of PDNVs is illustrated in Figure 4.

## 6 Challenges and future perspectives

### 6.1 Challenges

Plant-derived nanovesicles (PDNVs) demonstrate significant application potential in skin care. However, their development still faces numerous challenges. These challenges primarily concern isolation and purification technologies. They also relate to quality control, stability, safety assessment, and regulatory oversight. Regarding isolation and purification technologies, mainstream conventional methods have limitations. These methods include differential centrifugation and density gradient centrifugation. The limitations include complex operational procedures, long processing times, and low product yield. Furthermore, specific isolation and purification strategies often need to be established for nanovesicles derived from different plant sources, and there is currently a lack of universal, standardized operational protocols. Therefore, the development of novel, efficient, simple, and scalable isolation and purification technologies for PDNVs is one of the key research priorities in this field.

In terms of quality control, the composition of PDNVs is susceptible to influences. The biological properties are also susceptible. These influences come from multiple variables. These variables include plant variety, growth environment, harvesting time, and extraction processes. Consequently, significant batch-to-batch variations occur. Therefore, establishing scientific and unified quality control standards, covering key indicators such as particle size distribution, particle concentration, purity, and biological activity, is crucial for ensuring the stability and consistency of PDNV products. In terms of stability, PDNVs may undergo structural alterations and loss of biological activity during storage and application, thereby affecting their ultimate efficacy. Therefore, developing effective stabilizers and optimizing storage conditions to enhance the long-term stability of PDNVs is a prerequisite for their broad application. In terms of safety assessment, PDNVs are generally considered to have a high safety profile. However, potential risks exist. These risks are associated with long-term use and high-dose administration. They still require comprehensive evaluation through systematic studies. Therefore, establishing a comprehensive safety assessment system is essential. This system should cover key endpoint indicators. These indicators include cytotoxicity, genotoxicity, skin sensitization, and irritation. In terms of regulatory oversight, the regulatory framework for PDNVs as a novel bioactive ingredient in cosmetics, including classification, registration requirements, and filing management, remains incomplete. Therefore, promoting the development and refinement of relevant regulations and policies to provide clear legal basis and regulatory pathways for the application of PDNVs in cosmetics is a crucial condition for facilitating their healthy industrialization.

### 6.2 Future perspectives

Despite the a forementioned challenges, PDNVs still demonstrate broad application prospects and significant development potential in the field of skin care. At the level of basic research, future work will delve deeper into the biological functions and mechanisms of action of PDNVs, particularly the molecular mechanisms underlying their antioxidant, anti-inflammatory, anti-aging, and wound healing promotion effects. Simultaneously, in-depth research on the compositional and functional differences of PDNVs from different sources, as well as their interaction mechanisms with skin cells, will provide a solid theoretical foundation for precision skincare strategies based on PDNVs. At the technological innovation level, future research will be dedicated to developing novel, efficient, simple, and scalable isolation and purification technologies for PDNVs, such as microfluidic technology and affinity chromatography. Meanwhile, the development of novel PDNVs engineering technologies, including surface modification, targeting molecule conjugation, and even gene editing strategies, will significantly enhance the targeted delivery capability and therapeutic efficacy of PDNVs. In terms of application development, future research will focus on exploring nanovesicles from more plant sources and their combination applications to specifically address different skin concerns and care needs. Concurrently, exploring the synergistic effects between PDNVs and other active ingredients (such as traditional Chinese medicine extracts, natural plant essential oils, and synthetic drugs) and enhancing their therapeutic effects and application value through combination formulations is also an important direction. In terms of product innovation, future research will aim to develop novel cosmetic formulations based on PDNVs, such as PDNVs nanoemulsions, PDNV s gels, and PDNVs masks, to improve product stability, skin permeability, and bioavailability. Meanwhile, the development of intelligently responsive PDNV delivery systems (e.g., pH-sensitive, temperature-sensitive, photo-responsive) holds promise for achieving precise spatiotemporal control over the release and action of PDNVs. In terms of industrial development, future efforts will focus on establishing a comprehensive production and quality control system for PDNVs to promote their scaled production and commercial application. Simultaneously, strengthening industry-academia-research collaboration with enterprises, medical institutions, and research organizations will accelerate the industrialization and commercialization of PDNVs in the skin care field.

## 7 Conclusion

As a novel nanobiomaterial, PDNVs offer advantages such as wide source availability, cost-effectiveness, excellent biocompatibility, and low immunogenicity, and demonstrate significant efficacy in antioxidant, anti-inflammatory, anti-aging, and wound healing promotion activities. At the basic research level, the biological characteristics, isolation and identification techniques, and mechanisms of action of PDNVs have been relatively thoroughly investigated. Studies have shown that PDNVs can exert antioxidant, anti-inflammatory, anti-aging, and wound healing promotion effects through multiple pathways, providing a theoretical foundation for their skincare applications.

Looking forward, although the application of PDNVs in skin care faces multiple challenges including isolation and purification, quality control, stability, safety assessment, and regulatory oversight, their application prospects remain broad with continuous in-depth research and technological innovation. Future efforts should focus on developing efficient and scalable novel isolation and purification technologies, establishing scientific and unified quality control standards, improving the stability and safety of PDNVs, and promoting the refinement of relevant regulations and policies to facilitate their industrial development.

In summary, as a novel nano biomaterial, PDNVs exhibit significant application potential and development space in the field of skin care. With breakthroughs in basic research and technological advancements, PDNVs are expected to provide safer, more efficient, and more precise solutions for skin care, thereby promoting technological innovation and industrial development in this field.
